# An Examination of the Causes and Solutions to Eyewitness Error

**DOI:** 10.3389/fpsyt.2014.00102

**Published:** 2014-08-13

**Authors:** Richard A. Wise, Giuseppe Sartori, Svein Magnussen, Martin A. Safer

**Affiliations:** ^1^Department of Psychology, University of North Dakota, Grand Forks, ND, USA; ^2^Department of Psychology, University of Padua, Padua, Italy; ^3^Cognitive Developmental Research Unit, Department of Psychology, University of Oslo, Oslo, Norway; ^4^Department of Psychology, The Catholic University of America, Washington, DC, USA

**Keywords:** eyewitness, error, causes, solutions, legal safeguards

## Abstract

Eyewitness error is one of the leading causes of wrongful convictions. In fact, the American Psychological Association estimates that one in three eyewitnesses make an erroneous identification. In this review, we look briefly at some of the causes of eyewitness error. We examine what jurors, judges, attorneys, law officers, and experts from various countries know about eyewitness testimony and memory, and if they have the requisite knowledge and skills to accurately assess eyewitness testimony. We evaluate whether legal safeguards such as voir dire, motion-to-suppress an identification, cross-examination, jury instructions, and eyewitness expert testimony are effective in identifying eyewitness errors. Lastly, we discuss solutions to eyewitness error.

Researchers have been studying eyewitness testimony for over a century because of the frailties of human memory and the important role that it plays in wrongful convictions. Memory is a reconstructive process (1). Accordingly, post-event information supplied by the police, prosecutors, media, other eyewitnesses, family, and friends can alter not only an eyewitness’s memory of the crime but also the eyewitness’s memory of the perpetrator of the crime (2). For example, law officers can alter an eyewitness memory of the perpetrator by asking leading questions such as: “Did the perpetrator have blond hair?” Eyewitnesses are generally unaware that their memory of the crime or perpetrator has been altered by post-event information. Moreover, once an eyewitness memory is altered, it will be very difficult if not impossible to restore the eyewitness’s original memory of the crime or perpetrator (3).

Not only is eyewitness memory malleable, but so is eyewitness confidence. Many factors such as repeated questioning of eyewitness, confirming feedback (e.g., “Good, you have identified the suspect!”), and learning that another eyewitness has identified the same suspect can increase eyewitness confidence but not his or her accuracy. Because eyewitness confidence is generally the most important factor that the trier of fact uses in evaluating eyewitness accuracy, increases in eyewitness confidence can cause wrongful convictions (4). Factors that increase eyewitness confidence have their greatest effect on an eyewitness’s confidence for inaccurate information (5, 6).

Eyewitness error is a leading cause of wrongful convictions. For instance, eyewitness error was involved in about 75% of the 312 DNA exonerations cases in the U.S. (7). Gross and Shaffer (8) conducted a detailed analysis of 873 cases in the *National Registry of Exonerations*, a joint project of Michigan’s and Northwestern’s law schools, and determined that eyewitness misidentifications occurred in 667 (76%) cases. Smith and Cutler (9) analyzed 1198 cases of wrongful conviction and considered other factors relevant to the causes of wrongful convictions. They concluded “that about 50% of the cases of conviction of the innocent involved mistaken identification” (p. 11). Moreover, the American Psychological Association estimates that about one of every three eyewitnesses makes an erroneous identification (10).

In the following sections of this review, we examine what the principal participants in the criminal justice system and experts know about eyewitness testimony and memory, how effective legal safeguards are in detecting eyewitness error, and solutions that legal systems can implement to minimize eyewitness error.

## What the Principal Participants in Criminal Justice Systems and Experts Know about Eyewitness Testimony and Memory

### Jurors

If eyewitness error is going to be minimized, jurors and legal professionals must be knowledgeable about eyewitness factors and capable of applying them to the facts of a case. Studies of jurors and potential jurors in several different countries indicate that they have limited knowledge of eyewitness factors, though the extent of their knowledge varies, depending on the nature of the questions asked [(11) (U.S.); (12) (U.S.); (13) (Canada); (14) (Scotland); (15) (Norway); (16) (Australia); (17) (UK); (18) (U.S.)]. Furthermore, in assessing accuracy, jurors generally rely on factors that are poor predictors of accuracy, such as eyewitness confidence at trial, memory for minor details, and consistency of eyewitness testimony.

Jurors also tend to ignore factors that are good predictors of accuracy, such as whether the eyewitness wore a disguise, used a weapon, and most importantly the system variables in the case (19–21). System variables are eyewitness factors that generally the criminal justice system can control such as how the police conducted the eyewitness interviews and the identification procedures. Estimator variables are factors that cannot be controlled such as the lighting at the crime scene and whether the perpetrator wore a disguise (22). Lastly, jurors have trouble integrating their knowledge of eyewitness factors into the facts of a criminal case (23–26). Consequently, even if jurors were knowledgeable about eyewitness factors, they would still have difficulty assessing eyewitness accuracy. This result would likely occur because jurors would have problems applying their knowledge to the facts of a case.

### Judges

Psychologists have also studied what legal professionals and experts know about eyewitness testimony and memory. For example, Wise and Safer (27) surveyed 160 U.S. judges to determine their knowledge about eyewitness testimony, what they believe jurors know about eyewitness testimony, and what legal safeguards they would permit attorneys to use to educate jurors about eyewitness testimony. Eight of the questions in their survey were the same or similar to questions in a survey of eyewitness experts by Kassin et al. (28). The U.S. judges in the survey averaged only 55% correct on the 14-item knowledge scale. Moreover, many of the judges did not know key facts about eyewitness testimony such as the weak relationship between eyewitness confidence and accuracy at trial^1^, the important role that eyewitness error plays in wrongful convictions, and the difficulty jurors have in differentiating accurate and inaccurate eyewitnesses.

The U.S. judges’ responses differed significantly from the eyewitness experts’ responses on five of eight questions where they answered the same or similar questions. Compared to the eyewitness experts, the judges were more likely to believe that jurors were knowledgeable about eyewitness testimony. The judges in the survey were reluctant to permit the use of expert testimony to educate jurors about eyewitness testimony. Years of experience as an attorney or judge were not related to judges’ knowledge of eyewitness factors. In contrast, judges in the survey who were more knowledgeable about eyewitness testimony had many of the attitudes and beliefs that may be necessary to reduce eyewitness error. For example, they believed jurors have limited knowledge of eyewitness factors. They also had a greater willingness to permit the use of legal safeguards for eyewitness error including expert testimony.

Judges’ knowledge of eyewitness testimony has been assessed in other countries. For instance, Magnussen et al. (29) used a modified version of the Wise and Safer questionnaire to determine what 157 Norwegian judges knew about eyewitness testimony and compared their responses to the responses of the U.S. judges and the eyewitness experts of Kassin et al. (28). They found that the Norwegian judges’ responses were similar to the U.S. judges’ responses though they knew somewhat more about eyewitness testimony than the U.S. judges (63% correct vs. 55% correct).

As was true of the U.S. judges, the Norwegian judges knew significantly less about eyewitness testimony than the eyewitness experts and were less skeptical of jurors’ knowledge of eyewitness testimony than the experts. Similarly to the U.S. judges, Norwegian judges’ knowledge was not related to legal or judicial experience. Like the U.S. judges, greater knowledge of eyewitness testimony for the Norwegian judges was related to beliefs and attitudes that may be necessary to reduce eyewitness error. Other surveys of judges have produced similar results [(11, 30) (Swedish judges); (14) (Scottish judges); (31) (Chinese judges)]. Furthermore, it appears judges, like jurors, have difficulty integrating their knowledge of eyewitness testimony into the facts of a criminal case (32).

### Prosecutors and defense attorneys

A few studies have examined what defense attorneys and prosecutors know about eyewitness testimony. Most of these studies are out-of-date (33–36). There are, however, two recent surveys of attorneys’ knowledge of eyewitness testimony. Wise et al. (37) surveyed 73 prosecutors and 1184 defense attorneys in the U.S. about their knowledge of a wide array of estimator and system variables. The prosecutors averaged only 47% correct on the 13-item knowledge scale compared to 78% correct for the defense attorneys. The defense attorneys’ superior performance, however, appeared to result in part from their greater skepticism about eyewitness accuracy rather than their greater knowledge.

The prosecutors, compared to the defense attorneys, believed that jurors know significantly more about eyewitness testimony. The prosecutors were also significantly more likely than the defense attorneys to think that eyewitness testimony is reliable and were significantly less likely than the defense attorneys to believe that eyewitness error is a major factor in wrongful convictions. The prosecutors were significantly less willing than the defense attorneys to permit the use of legal safeguards to educate jurors about eyewitness testimony, including eyewitness expert testimony. Moreover, like the judges, attorneys’ knowledge of eyewitness testimony was not related to legal experience, but was related to attitudes and beliefs that may be necessary to reduce eyewitness error.

In another recent survey, Magnussen et al. (38) evaluated what 100 Italian defense attorneys know about eyewitness testimony. The Italian defense attorneys averaged 71% correct on a 12-item knowledge scale. Similar to the results reported in the survey of the U.S. defense attorneys, the Italian defense attorneys’ superior performance appeared to result at least in part from their greater skepticism of eyewitness testimony rather than their greater knowledge. Like prior surveys of legal professionals, greater knowledge for the Italian defense attorneys was not related to legal experience but was related to attitudes and beliefs that may be necessary to reduce eyewitness error. Finally, as was true of jurors and judges, attorneys appear to have difficulty applying their knowledge of eyewitness factors to the facts of a case (32).

### Law officers

If legal systems are going to minimize eyewitness error, law officers must identify the relevant eyewitness factors at the crime scene and conduct proper eyewitness interviews and identification procedures. Moreover, because memory is a reconstructive process, once law officers conduct a biased eyewitness interview or identification procedure they generally cannot correct their errors by subsequently conducting proper procedures (39).

In 1999, the National Institute of Justice (“NIJ”), the research arm of the U.S. Department of Justice, published *Eyewitness Evidence: A Guide for Law Enforcement* (“*Guide*”). The *Guide* contained recommended procedures for U.S. law (40) enforcement agencies for conducting eyewitness interviews and identification procedures. The *Guide* was heavily based on scientific research on eyewitness testimony. In 2003, NIJ published *Eyewitness Evidence: A Training Manual for Law Enforcement* (“*Training Manual*”) (41), which provided training in the *Guide’s* procedures for law enforcement agencies.

Surveys of law officers in various countries show that they have limited knowledge of eyewitness factors [(11) (U.S.); (42) (Canada); (30) (Sweden); (43) (Estonia); (39) (U.S.)]. For example, Wise et al. (39) surveyed 532 U.S. law officers. Their sample included 83 officers from jurisdictions that had implemented eyewitness reforms, and 449 officers from jurisdictions that had not implemented eyewitness reforms. The reform officers averaged 6.11 (56%) correct on the 11-item knowledge scale about eyewitness factors while the non-reformed officers averaged 52% (5.68) correct answers. Similarly in the survey by Fraser et al. (42) of 168 Canadian law officers, they averaged 8.55 (61.1%) correct answers on the survey’s 14-item knowledge scale about eyewitness factors.

Wise et al. (39) also found that both the reform officers and the non-reform officers failed to follow many of the *Guide’s* procedures for conducting eyewitness interviews, photo lineups, and show-ups. Of the 17 *Guide* procedures in their survey for conducting eyewitness interviews, lineups, and show-ups, the reform officers reported following on average only 12.21 (72%) of the procedures. The non-reform officers reported following on average, only 11.06 (65%) of the *Guide’s* procedures. Moreover, only 18% of the reform officers and only 1% of the non-reform officers in the survey had both read the *Guide* and received training in its procedures.

The Police Executive Research Forum (44) conducted the first nationwide assessment of how U.S. law enforcement agencies conduct identification procedures. The survey showed that U.S. law enforcement agencies lack uniformity in their identification procedures and that most agencies have not fully implemented the *Guide’s* procedures. In fact, some *Guide* procedures have been implemented in less than half of the agencies, and the majority of the U.S. law enforcement agencies still use non-blind, simultaneous lineup procedures.

### Expert witnesses

Magnussen and Melinder (45, 46) surveyed 858 licensed Norwegian psychologists about their knowledge and beliefs about memory. Their results were compared to prior surveys of legal professionals and lay persons and to scientific studies of memory. The psychologists were no more knowledgeable about eyewitness memory than judges or lay persons, and a substantial minority of the psychologists had scientifically unsupported beliefs about memory. In a follow-up study, Melinder and Magnussen (47) determined if 115 Norwegian psychiatrists and psychologists who had testified as expert witnesses in court were more knowledgeable about memory than 819 Norwegian psychiatrists and psychologists who had never been an expert witness. Contrary to their expectations, the psychiatrists and psychologists who testified as expert witnesses were no more knowledgeable about memory than the psychiatrists and psychologists who had never been an expert witness.

This survey also included a few questions about memory that are not only relevant to criminal cases but also to the practice of psychiatry and psychology. These questions asked respondents how young children’s memory compares to adult memory, how memory for traumatic events compares to memory for mundane events, and whether childhood and adult traumatic experiences can be repressed. Accordingly, the questions covered information that psychiatrists and psychologists should know about memory, even if they do not testify as expert witnesses. Once again, a substantial minority of the psychiatrists and psychologists, including those who testified as expert witnesses, had scientifically unsupported beliefs about memory. Mirandola et al. (48) reported very similar results when they surveyed psychology students and psychology professors at a major Italian university. A recent study showed that many U.S. psychologists also have scientifically unsupported beliefs about memory (49). Melinder and Magnussen (47) concluded from their surveys that expert witnesses are at risk of providing courts with faulty information about memory.

### Conclusion

The principal participants in criminal justice systems appear to have limited knowledge of eyewitness factors. Moreover, it is likely that surveys overestimate their knowledge. Surveys focus respondents’ attention on the relevant eyewitness factors, only require respondents to recognize rather than generate the correct answer, and usually do not take guessing into account. For example, Houston et al. (14) determined that jury eligible participants produced significantly more correct answers when they completed a multiple choice questionnaire about eyewitness factors than when they had to produce their own responses to questions.

Alonzo and Lane (50) found that respondents’ answers to surveys about eyewitness factors did not predict how they assessed eyewitness accuracy. This result is not surprising. As previously stated, research shows that the principal participants in criminal justice systems have trouble applying their knowledge of eyewitness factors to the facts of a case. Consequently, even if they were knowledgeable about eyewitness factors, they would probably still have problems assessing eyewitness accuracy. As Cutler and Penrod (51) stated, even experts have difficulty integrating their knowledge into the facts of a case. In sum, legal systems should be alarmed that not only jurors, but legal professionals and even many experts appear to lack the necessary knowledge and skills to accurately assess eyewitness testimony.

## Legal Safeguards

Because the principal participants in criminal justice systems lack the requisite knowledge and skills to accurately assess eyewitness testimony, legal systems need safeguards that either prevent erroneous eyewitness identifications from being introduced into evidence at trial or enable the trier of fact to accurately assess eyewitness testimony. The U.S. legal system employs several safeguards in attempting to achieve these vital goals.

## Motion-To-Suppress an Identification

A defense attorney in the U.S. can file a motion-to-suppress an identification to prevent a suggestive identification from being introduced at trial. For a motion-to-suppress to be an effective safeguard, several requirements must be met. First, defense attorneys must be present at identification procedures so they can determine if they were suggestive. Second, attorneys and judges must know the factors that cause suggestive identification procedures. Third, they must be capable of applying their knowledge to identification procedures.

Defense attorneys are usually not present at identification procedures in the U.S. because photo arrays are primarily used for identifications rather than live lineups (52). In the U.S., there is no right to have an attorney present at a photo array even after an indictment (53). In addition, as was previously stated, attorney and judges have limited knowledge of eyewitness factors and have difficulty applying them to the facts of a case.

## Voir Dire

Voir dire is the process of selecting a jury in the U.S. If attorneys can select jurors who will critically and carefully evaluate eyewitness testimony, voir dire could be an effective remedy for eyewitness error. For voir dire to be an effective legal safeguard, two requirements must be met. Attorneys must have sufficient opportunity to assess potential jurors’ attitudes and beliefs about eyewitness testimony during voir dire. Second, attorneys must be capable of making these assessments (52). In some U.S. courts, attorneys can extensively question potential jurors. In other courts, attorneys’ questioning of potential jurors is very limited or may even be non-existent.

Narby and Cutler (54) developed the Attitudes to Eyewitness Scale to assess jurors’ attitudes and beliefs about eyewitness testimony. It was successful in only one of three studies in predicting how mock jurors assess eyewitness testimony (52). In short, because attorneys’ lack the opportunity in many U.S. courts to assess jurors’ attitudes and beliefs about eyewitness testimony and because they lack a valid instrument for assessing them, it does not appear that voir dire is an effective remedy for eyewitness error.

## Cross-Examination

For cross-examination to be an effective safeguard, three conditions must be met. First, attorneys must be knowledgeable about eyewitness factors so they can identify for jurors during cross-examination the factors that indicate that the eyewitness may have made an erroneous identification. Second, jurors must be knowledgeable about eyewitness factors so they can comprehend the significance of the cross-examination. Lastly, jurors must be capable of applying the relevant eyewitness factors revealed during cross-examination to the facts of a case (52). As previously discussed, attorneys and jurors have limited knowledge of eyewitness factors, and they have difficulty applying their knowledge to the facts of a case.

## Jury Instructions

Because of the ineffectiveness of other safeguards, many scholars recommend that courts use jury instructions or expert testimony to sensitize jurors to eyewitness testimony. Unfortunately, the limited research conducted on jury instructions shows that they do not protect against eyewitness error. Several studies tested the Telfaire jury instructions. The Telfaire instructions are eyewitness jury instructions that the U.S. Court of Appeals for the District of Columbia created in Telfaire (55). The Telfaire instructions are the most widely used eyewitness jury instructions in the U.S (56, 57). The Telfaire instructions include five factors that jurors should consider when evaluating eyewitness accuracy and are based on the U.S. Supreme Court’s decisions in Biggers (58) and Braithwaite (59)^2^.

Studies of the Telfaire instructions uniformly show that they do not sensitize jurors to eyewitness testimony (56, 57, 60, 61). Because of their ineffectiveness, psychologists have attempted to improve them. For example, Greene (57) simplified and clarified the Telfaire instructions. She also added to the Telfaire instructions information about the effects of certain eyewitness factors on accuracy such as eyewitness confidence, stress, and the retention interval. Greene found that her revised eyewitness instructions increased mock-juror skepticism, but did not increase their sensitivity to eyewitness testimony. Ramirez et al. (61) also tried to enhance their effectiveness by simplifying them and including 13 eyewitness factors that commonly affect eyewitness accuracy. The revised instructions failed to increase mock-jurors’ sensitivity to eyewitness testimony.

Bornstein and Hamm (56) tried various means to increase the efficacy of eyewitness jury instructions. They simplified and modified them to include specific factors relevant to accuracy. They provided mock jurors with written jury instructions as well as verbal instructions. They manipulated the judge’s demeanor when presenting the jury instructions and created interactive jury instructions. None of their modifications increased jurors’ sensitivity. They concluded: “… the research on modifying instructions about witness identification has generally failed to accomplish this goal [of sensitizing jurors], and the present studies do not afford a much more optimistic conclusion” (p. 53). Lastly, jury instructions from Henderson (62), which are based on eyewitness research, are also ineffective in sensitizing jurors to eyewitness testimony (63).

## Eyewitness Expert Testimony

Cutler et al. (64) stated that eyewitness expert testimony can produce three effects: (1) no effect because the trier of fact does not understand the expert testimony or is not persuaded by it; (2) enhanced skepticism, which causes the trier of fact to disbelieve all eyewitnesses no matter how good the eyewitness conditions; and (3) enhanced sensitivity, which educates the trier of fact about eyewitness factors and how to apply them to the facts of the case. Clearly, the desirable effect of expert testimony or any other legal safeguard is to increase the trier of fact’s sensitivity to eyewitness testimony.

The most common effect of eyewitness expert testimony is to increase jurors’ skepticism of eyewitnesses. For example, Leippe and his colleagues have summarized on several occasions the literature on eyewitness expert testimony (65–67). In their most recent summary, they divided the research on eyewitness expert testimony into two categories: studies prior to 1995 and studies after 1995 (66). They found 12 studies of expert testimony prior to 1995. In 10 of the 12 studies, expert testimony increased juror skepticism. In reviewing eight of these studies, where it could be evaluated if expert testimony increased juror sensitivity to eyewitness factors, they found that only two studies increase sensitivity. They concluded: “Sensitivity appeared to be more the exception than the rule” (p. 176).

Leippe and Eisenstadt (66) discussed seven additional studies of eyewitness expert testimony that were conducted after 1995. These studies further demonstrated the difficulty that expert testimony has in sensitizing the trier of fact to eyewitness factors. In the latest study of eyewitness expert testimony, Martire and Kemp (68) determined if it could sensitize participants to the relationship between eyewitness confidence and accuracy. They found that, though the participants seemed to understand what the expert said about the relationship between eyewitness confidence and accuracy, they did not integrate their knowledge into their assessments of eyewitness accuracy. In sum, there is no consistently effective legal safeguard for eyewitness error.

## Solutions to Eyewitness Error

### Education

Because the principal participants in criminal justice systems have limited knowledge of eyewitness factors, it is essential that legal systems educate them about eyewitnesses. Law schools should teach law students about eyewitnesses. For example, law courses such as criminal law and criminal procedure could include in-depth information about the different types of eyewitness error, the causes of eyewitness error, and the legal safeguards needed to minimize eyewitness error. Judges’ and attorneys’ training should include extensive instruction about eyewitnesses. Law enforcement agencies need to incorporate detailed information about eyewitnesses when they train law officers. Professional organizations should offer courses about eyewitnesses for psychologists and psychiatrists who testify about it.

Moreover, psychologists who conduct research on eyewitness testimony should be knowledgeable about criminal investigations and trials so their recommendations for eyewitness reforms comport with the realities of a criminal justice system. Wilford and Wells (69) cite examples of impractical reforms eyewitness researchers have proposed. For instance, some researchers recommend that law enforcement agencies do not use show-ups because they are more suggestive than lineups. In fact, show-ups are essential to U.S. law enforcement because they allow law officers to arrest suspects shortly after a crime.

Courts could provide information about eyewitnesses to potential jurors who may hear criminal cases. Furthermore, legal professionals and experts need periodic refresher courses to keep them informed about the latest developments in eyewitness research. Legislation mandating that legal professionals receive eyewitness training may be the most effective means to accomplish this essential goal.

As previously stated, accurate assessment of eyewitness accuracy requires both knowledge of eyewitness factors and the ability to apply them to the facts of the case. Accordingly, courses for legal professionals, psychiatrists, and psychologists should not only educate them about memory, eyewitness factors, and proper procedures for eyewitness interviews and identification procedures; but also teach them how to integrate their knowledge into the facts of a case. Because of their expertise in eyewitness testimony and assessment, psychologists should work with legal professionals to develop courses about eyewitnesses and help legal professionals assess the courses’ reliability and validity.

### Proper eyewitness interviews and identifications

The most potent means available to the legal system to reduce eyewitness error is to conduct proper eyewitness interviews and identification procedures. It is much easier to prevent eyewitness errors than to detect them once they have occurred (69). We have seen, however, that despite NIJ promulgating the *Guide* and *Training Manual*, U.S. law enforcement agencies have been slow in adapting eyewitness reforms that are necessary for proper procedures. Moreover, law enforcement must stay informed about new research on proper procedures. This means law enforcement needs to regularly review their procedures for eyewitness interviews and identification procedures to determine if they need revision.

Because conducting proper eyewitness interviews and identification procedures is essential to reducing eyewitness error, legal systems should view eyewitness evidence as a type of trace evidence, like DNA or blood evidence (70). Consequently, the use of scientific procedures in producing eyewitness evidence should be an important factor in determining whether eyewitness evidence is admitted in criminal cases. In addition, like other types of trace evidence, legal systems should generally require law officers who collect eyewitness evidence to be trained and certified in scientific procedures for conducting eyewitness interviews and identification procedures.

More pressure needs to be exerted on legal systems to institute proper eyewitness interviews and identification procedures. Potential sources of influence on legal systems to implement proper procedures include legislation, court decisions, expert testimony, and media attention about the problem of eyewitness error (69).

### Improved legal safeguards

Even if law enforcement adapted all the necessary reforms for proper interviews and identification procedures, eyewitness errors would still occur. As we have seen, legal professionals and jurors have difficulty assessing eyewitness accuracy and currently no legal safeguard consistently sensitizes the trier of fact to eyewitness factors. Accordingly, if legal systems are going to minimize eyewitness error, they must develop an effective safeguard for identifying it.

To address this problem, Wise created the interview-identification-eyewitness factor (I-I-Eye) method for analyzing eyewitness accuracy (71, 72). The I-I-Eye method consists of four steps: first, you determine if law officers (a) conducted the eyewitness interviews so they obtained the maximum amount of accurate information, (b) did not contaminate the eyewitness’s memory of the crime with post-event information, or (c) artificially increased the eyewitness’s confidence. In the second step, you assess if the identification procedures in the case were properly conducted. During the third step, you evaluate how the eyewitness factors during the crime likely affected eyewitness’s accuracy. In the fourth step, you make conclusions about the likely accuracy of the eyewitness testimony in the case.

Three studies testing the I-I-Eye method showed that it sensitizes jurors to eyewitness testimony [(21, 73); Wise and Kehn (under review)]. Although the results so far have been encouraging, much more research is needed before it can be concluded that I-I-Eye method is an effective safeguard for eyewitness error.

As was previously discussed, there has been limited research on improving legal safeguards. Developing effective legal safeguards for detecting eyewitness error should be a top priority for eyewitness researchers and legal professionals.

### Greater cooperation with legal professionals and more field studies of eyewitness reforms

Psychologists should work closely with legal professionals in developing and testing eyewitness reforms. Legal professionals have expertise, knowledge, and experience that psychologists lack, and their skills and knowledge are essential to creating effective reforms that have strong ecological validity. Furthermore, legal professionals must deal with the adverse consequences of eyewitness reforms such as administrative difficulties in implementing them, increased costs, and fewer accurate eyewitness identifications. Therefore, it is vital that legal professionals are involved in developing and testing eyewitness reforms so they are motivated to successfully implement them despite their problems.

Many law officers, prosecutors, and even some judges view eyewitness reforms with suspicion because they believe that they only benefit the defense, will primarily result in guilty defendants going free, and do not take into account the realities of a criminal justice system. Accordingly, psychologists need to do a better job of educating legal professionals how reforms can benefit them (69). For example, conducting proper eyewitness interviews and identification procedures substantially strengthen prosecutors’ cases, help alleviate increasing juror concerns about the reliability of eyewitness testimony, and reduce defendants’ use of eyewitness expert testimony.

Psychologists should also conduct more field studies to ensure that eyewitness reforms have strong ecological validity. The Hennepin County’s blind sequential pilot project (74) and the Greensboro Protocol (75) are excellent examples of well-conducted field studies about eyewitness testimony. They also demonstrate the great progress that can be made in eyewitness research when psychologists and legal professionals work together to reduce eyewitness error.

## Conclusion

Wrongful convictions are not only a tragedy for innocent defendants and their families but also for the victims of additional crimes that occur because the true perpetrator of a crime has not been apprehended. Moreover, wrongful convictions undermine the public’s faith in the law especially when the wrongful convictions are preventable (27). The solutions discussed above could help to significantly reduce eyewitness error and the wrongful convictions that result from them.

## Conflict of Interest Statement

The authors declare that the research was conducted in the absence of any commercial or financial relationships that could be construed as a potential conflict of interest.

## Footnotes

^1^In both the U.S. and the Norwegian survey of judges, approximately one-third of the judges thought that at trial eyewitness confidence is related to accuracy, one-third thought that it is not related to accuracy, and one-third did not know. These results suggest that generally judges are unaware that at trial, eyewitness confidence is a poor predictor of accuracy. This finding is particularly troubling because as previously stated eyewitness confidence is the most important factor the trier of fact generally uses in evaluating eyewitness accuracy. Accordingly, because of their lack of knowledge, judges are unlikely to correct jurors’ erroneous belief that eyewitness confidence at trial predicts accuracy.

^2^The five eyewitness factors discussed in the Telfaire instructions are: (1) the quality of the eyewitness’ view of the perpetrator of the crime; (2) the time between the crime and the identification procedure; (3) the eyewitness’s confidence in the accuracy of the identification; (4) the accuracy of the eyewitness’ description of the perpetrator; and (5) the degree of attention the eyewitness paid to the crime. These factors have limited utility in assessing eyewitness accuracy (76).
